# Measuring the Burden of Infodemics: Summary of the Methods and Results of the Fifth WHO Infodemic Management Conference

**DOI:** 10.2196/44207

**Published:** 2023-02-20

**Authors:** Elisabeth Wilhelm, Isabella Ballalai, Marie-Eve Belanger, Peter Benjamin, Catherine Bertrand-Ferrandis, Supriya Bezbaruah, Sylvie Briand, Ian Brooks, Richard Bruns, Lucie M Bucci, Neville Calleja, Howard Chiou, Abhinav Devaria, Lorena Dini, Hyjel D'Souza, Adam G Dunn, Johannes C Eichstaedt, Silvia M A A Evers, Nina Gobat, Mika Gissler, Ian Christian Gonzales, Anatoliy Gruzd, Sarah Hess, Atsuyoshi Ishizumi, Oommen John, Ashish Joshi, Benjamin Kaluza, Nagwa Khamis, Monika Kosinska, Shibani Kulkarni, Dimitra Lingri, Ramona Ludolph, Tim Mackey, Stefan Mandić-Rajčević, Filippo Menczer, Vijaybabu Mudaliar, Shruti Murthy, Syed Nazakat, Tim Nguyen, Jennifer Nilsen, Elena Pallari, Natalia Pasternak Taschner, Elena Petelos, Mitchell J Prinstein, Jon Roozenbeek, Anton Schneider, Varadharajan Srinivasan, Aleksandar Stevanović, Brigitte Strahwald, Shabbir Syed Abdul, Sandra Varaidzo Machiri, Sander van der Linden, Christopher Voegeli, Claire Wardle, Odette Wegwarth, Becky K White, Estelle Willie, Brian Yau, Tina D Purnat

**Affiliations:** 1 US Centers for Disease Control and Prevention Atlanta, GA United States; 2 Brazilian Inmunization Society São Paulo-SP Brazil; 3 Department of Political Science and International Relations Université de Genève Geneva Switzerland; 4 HealthEnabled Cape Town South Africa; 5 Olylo Paris France; 6 Department of Epidemic and Pandemic Preparedness and Prevention World Health Organization Geneva Switzerland; 7 Center for Health Informatics School of Information Sciences University of Illinois Champaign, IL United States; 8 Johns Hopkins Center for Health Security Baltimore, MD United States; 9 Immunize Canada Canadian Public Health Association Ottawa, ON Canada; 10 Directorate for Health Information and Research Ministry for Health Valletta Malta; 11 US Public Health Service Commissioned Corps Rockville, MD United States; 12 The George Institute for Global Health New Delhi India; 13 Working Group Health Policy and Systems Research and Innovation Institute for General Practice Charité Universitätsmedizin Berlin Berlin Germany; 14 Biomedical Informatics and Digital Health Faculty of Medicine and Health University of Sydney Sydney Australia; 15 Department of Psychology Stanford University Stanford, CA United States; 16 Institute for Human-Centered AI Stanford University Stanford, CA United States; 17 Department of Health Services Research Maastricht University Maastricht Netherlands; 18 Department of Country Readiness Strengthening World Health Organization Geneva Switzerland; 19 Department of Knowledge Brokers THL Finnish Institute for Health and Welfare Helsinki Finland; 20 Field Epidemiology Training Program Epidemiology Bureau Department of Health Manila Philippines; 21 Ted Rogers School of Management Toronto Metropolitan University Toronto, ON Canada; 22 Department of Epidemiology and Biostatistics Graduate School of Public Health and Health Policy City University of New York New York, NY United States; 23 Department Technological Analysis and Strategic Planning Fraunhofer Institute for Technological Trend Analysis INT Euskirchen Germany; 24 Infection Prevention and Control Department Children's Cancer Hospital Egypt-57357 Ain Shams University Specialized Hospital Cairo Egypt; 25 Department of Social Determinants World Health Organization Geneva Switzerland; 26 European Healthcare Fraud and Corruption Network Aristotle Universtity of Thessaloniki Brussels Belgium; 27 Global Health Program Department of Anthropology University of California San Diego, CA United States; 28 Institute of Social Medicine Faculty of Medicine University of Belgrade Belgrade Serbia; 29 Observatory on Social Media Luddy School of Informatics, Computing, and Engineering Indiana University Bloomington, IN United States; 30 DataLEADS (Health Analytics Asia) New Delhi India; 31 Technology and Social Change Project Harvard University Cambridge, MA United States; 32 Health Innovation Network Guy’s and St Thomas’ Hospital London United Kingdom; 33 Center of Science and Society Columbia University New York, NY United States; 34 Instituto Questão de Ciência São Paulo Brazil; 35 Department of Health Services Research Care and Public Health Research Institute Maastricht University Maastricht Netherlands; 36 Clinic of Social and Family Medicine Faculty of Medicine University of Crete Heraklion Greece; 37 American Psychological Association Washington DC, DC United States; 38 Department of Psychology and Neuroscience University of North Carolina at Chapel Hill Chapel Hill, NC United States; 39 Department of Psychology University of Cambridge Cambridge United Kingdom; 40 Bureau for Global Health Office of Infectious Disease United States Agency for International Development Washington DC, DC United States; 41 Pettenkofer School of Public Health Ludwig-Maximilians-Universität München Munich Germany; 42 Graduate Institute of Biomedical Informatics Taipei Medical University Taipei Taiwan; 43 African Field Epidemiology Network Harare Zimbabwe; 44 Office of the Director National Center for Immunization and Respiratory Diseases US Centers for Disease Control and Prevention Atlanta, GA United States; 45 Information Futures Lab School of Public Health Brown University Providence, RI United States; 46 Heisenberg Chair for Medical Risk Literacy & Evidence-Based Decisions Charite – Universitätsmedizin Berlin Berlin Germany; 47 Communications, Policy, Advocacy The Rockefeller Foundation New York, NY United States

**Keywords:** COVID-19, infodemic, burden of infodemic, infodemic management, infodemic metrics, World Health Organization, technical consultation, infodemiology

## Abstract

**Background:**

An infodemic is excess information, including false or misleading information, that spreads in digital and physical environments during a public health emergency. The COVID-19 pandemic has been accompanied by an unprecedented global infodemic that has led to confusion about the benefits of medical and public health interventions, with substantial impact on risk-taking and health-seeking behaviors, eroding trust in health authorities and compromising the effectiveness of public health responses and policies. Standardized measures are needed to quantify the harmful impacts of the infodemic in a systematic and methodologically robust manner, as well as harmonizing highly divergent approaches currently explored for this purpose. This can serve as a foundation for a systematic, evidence-based approach to monitoring, identifying, and mitigating future infodemic harms in emergency preparedness and prevention.

**Objective:**

In this paper, we summarize the Fifth World Health Organization (WHO) Infodemic Management Conference structure, proceedings, outcomes, and proposed actions seeking to identify the interdisciplinary approaches and frameworks needed to enable the measurement of the burden of infodemics.

**Methods:**

An iterative human-centered design (HCD) approach and concept mapping were used to facilitate focused discussions and allow for the generation of actionable outcomes and recommendations. The discussions included 86 participants representing diverse scientific disciplines and health authorities from 28 countries across all WHO regions, along with observers from civil society and global public health–implementing partners. A thematic map capturing the concepts matching the key contributing factors to the public health burden of infodemics was used throughout the conference to frame and contextualize discussions. Five key areas for immediate action were identified.

**Results:**

The 5 key areas for the development of metrics to assess the burden of infodemics and associated interventions included (1) developing standardized definitions and ensuring the adoption thereof; (2) improving the map of concepts influencing the burden of infodemics; (3) conducting a review of evidence, tools, and data sources; (4) setting up a technical working group; and (5) addressing immediate priorities for postpandemic recovery and resilience building. The summary report consolidated group input toward a common vocabulary with standardized terms, concepts, study designs, measures, and tools to estimate the burden of infodemics and the effectiveness of infodemic management interventions.

**Conclusions:**

Standardizing measurement is the basis for documenting the burden of infodemics on health systems and population health during emergencies. Investment is needed into the development of practical, affordable, evidence-based, and systematic methods that are legally and ethically balanced for monitoring infodemics; generating diagnostics, infodemic insights, and recommendations; and developing interventions, action-oriented guidance, policies, support options, mechanisms, and tools for infodemic managers and emergency program managers.

## Introduction

### The Challenge That Infodemics Pose to Health System Response in Emergencies

An infodemic is excess information of varying quality, including false/misleading information or ambiguous information or both, that spreads in digital and physical environments during a health emergency [1,2]. Infodemics are more complex than just the amplification and spread of mis- and disinformation; they spread across a wider information landscape where population questions, concerns, and information voids can lead to misinformation growth and spread, particularly in societies undergoing digital transformation. The COVID-19 pandemic has been accompanied by an unprecedented global infodemic that has led to confusion about the benefits of medical and public health interventions, with substantial impact on risk-taking and health-seeking behaviors, eroding trust in health authorities and compromising the effectiveness of public health responses and policies [3].

There are several key concepts that are integral to discussing infodemics and how they link to health authority responses, including the online information environment; the channels, formats, and quality of health information people are exposed to; individual-level literacy; the psychology of emergencies; and the multifaceted aspects of trust and how they influence perception and behavior in health. Many of these areas have bodies of research and literature and measures associated with them in specific fields of study, such as psychology, governance and policy, and digital user experience, but they are usually not connected in a systematic, causal way that is applicable to how health systems act in emergencies.

### The Information Environment and Accessing Health Information

As the world becomes more digitized, the digital information environment increasingly influences social dynamics between people and across communities, influencing health decisions and behaviors [4-6]. The accessibility and availability of health information, the algorithms of social media platforms, the architecture of online communities and news channels, and format all impact how individuals receive and act on health information [4,6]. Creating and updating credible, accurate health information for dissemination to different audiences is within the purview of health authorities and tends to be formalized in policies for health care delivery and in public health matters, especially in emergencies [7]. However, health information is often shared through unofficial and unregulated channels and made available in a wider variety of formats and for channels not typically used by the health system or for communication of public health guidance, creating a gap between which communities have access to official and credible information and those that do not [8]. For example, TikTok and closed messaging networks, such as WhatsApp and Telegram, have increasingly been used to share health information and misinformation [9].

### Literacies Related to Health, Infodemics, and Emergencies

Simply having access to health information is insufficient for instigating positive behavior change [10]. Health, digital, media, science, information, and influence literacies all play a role at the individual level, mediating between the availability of health information and the individual ability to process, understand, and act on it [11]. However, in emergencies, people seek, process, and act on information differently, looking for information to protect themselves and their families, even though information may be scarce, and looking for alternate sources of information, while tending to believe the first thing they hear. There are examples related to noncommunicable diseases, such as tobacco cessation campaigns, that aim to address health literacy gaps and counter harm from misinformation [12]. Teaching critical thinking skills to individuals to be able to identify and rebut health misinformation can broadly inoculate against specific misinformation narratives and is one promising intervention for building resilient individuals and communities against misinformation. Therefore, building skills and resilience against misinformation and other infodemic harms and improving the use of health information during times of calm are not sufficient alone to help people during emergencies, where traditional health communications pathways, such as communicating with a primary health care provider, may have been interrupted.

### Building Trust to Prevent Erosion During Emergencies

Building trust in health authorities is critical before emergencies strike, because infodemics can quickly erode trust, especially when there is low trust at baseline. Trust contributes toward the willingness to accept and adopt necessary measures and can be the deciding factor in terms of how successful the implementation of a sound public health strategy will be—for example, in the context of implementing public health and social measures to control a disease as part of containment strategies. Trust can be eroded by what the public may perceive as conflicting guidance and mixed messages, information released late, multiple experts with divergent opinions, paternalism, and political infighting [13]. Infodemics can further add friction by promoting misinformation and more destructive forms, such as disinformation or conspiracy theories; not addressing people’s questions and concerns in a timely manner; or leaving people struggling to access accurate, credible, and up-to-date health information [14].

### Why Measure the Burden and Cost of Infodemics?

Due to the multifaceted nature of infodemics affecting individuals, communities, societies, economies, and health systems during emergencies, it can be difficult to know how to prepare for infodemics, determine when and where to intervene, and understand how to more effectively reduce harm to population health. Globally applicable infodemic measurements and metrics are needed. The true cost of infodemics has not been robustly measured but has been anecdotally reported, with impacts such as stigma, violence against health workers, overdoses of nonrecommended treatments or stockouts, refusal by individuals or communities to wear masks or get vaccinated, and frivolous lawsuits against health systems and health care workers [15]. One academic brief suggested that COVID-19 misinformation cost US $50-$300 million a day at the height of the pandemic in the United States [16]. Without measures or costing, it is difficult to develop effective interventions and advocate for supportive policies. More innovation in how measurements and metrics are developed is needed due to the multilevel nature of the phenomenon and the sheer diversity of disciplines and in-depth expertise required to measure or estimate different aspects of infodemics.

### Spurring the Development of Metrics to Measure the Burden of Infodemics and Interventions as Part of the WHO Public Health Research Agenda on Infodemiology

Since the beginning of the COVID-19 pandemic, the World Health Organization (WHO) has expanded the concept of infodemiology beyond the use of data produced and consumed on the web to inform public health officials, agencies, and policy into a multidisciplinary scientific field. Interventions must account for an information environment where information flows online and offline, highly tailored to people’s information diets, and their responses can lead to nonprotective behaviors and poor health outcomes offline [1,2,17]. Building harmonized measures and cohesive interventions requires an amalgamation of cross-disciplinary and mixed methods approaches to inform the health emergency response and routine health system–strengthening efforts online and offline [17].

Early in the COVID-19 response, the First WHO Infodemiology Conference in June-July 2020 brought together experts from a range of disciplines to begin a global conversation about the science of infodemiology and establish a public health research agenda for managing infodemics, recognizing that each discipline has a different perspective on the problems of infodemics, different ways of measurement, and a different vocabulary [17,18]. Although previous conferences have expanded our understanding of infodemic drivers [19] and social listening approaches [20], the Fifth WHO Infodemic Management Conference aimed to collaboratively develop a proposed action plan to foster implementation for work stream 1 of the WHO public health research agenda for managing infodemics: the development of metrics and indicators for measuring the burden of an infodemic and related interventions. The full conference report is available on the WHO website [21]. In this paper, we summarize the conference structure, proceedings, outcomes, and proposed actions.

## Methods

### Overview

The conference used an iterative human-centered design (HCD) approach in line with the purpose-outcome-process (POP) model, a tool for focusing actions on creating results [22,23]. Held in the context of the COVID-19 pandemic and with travel restrictions in place, the meeting necessarily took place online via videoconference. The virtual discussions took place over four 3-hour meetings during 2 weeks in November 2021, resulting in a summary report and recommended actions to advance 5 key areas for the development of metrics to assess the burden of infodemics. The summary report consolidated the participants’ input for a common vocabulary, concepts, standardized study designs, measures, and tools to estimate the burden of infodemics or the effectiveness of infodemic management interventions.

### Ethical Considerations

Institutional Review Board review was not sought because the work described in this paper was based on observation of discussions at the conference and focused on the synthesis of expert opinion following the Chatham House Rule [24]. No personal information was collected from participating experts.

### Design Approaches to Promote Effective Interdisciplinary Discussion

The organizers used an HCD approach to intentionally facilitate engaging and effective conference deliberations [23]. First, the conference format was designed to offer a level playing field for all participants who were encouraged to contribute their knowledge in an environment where most participants came from extremely diverse disciplines, backgrounds, country settings, and professional experiences. Second, the conference structure was designed with the help of the POP model [22] to provide a structured output to conference deliberations. Third, sessions were scheduled on different days, allowing the organizing team to synthesize inputs and prepare for the next session and adapt the deliberations and format. Consideration was given to what and how essential information was shared with participants before and during the conference sessions, to the emotional pacing of the interactions that would support intense cross-disciplinary expert deliberations, and to the environment that would support the participant behaviors and discussions toward actionable recommendations.

#### First Approach: Designing for Consensus on Outcomes and Recommendations

The 4 conference sessions were structured over the following thematic areas for a cumulative duration of 12 hours. Facilitators directed participant discussions to arrive at actionable recommendations on the last day. Ahead of each session, the design of the proceedings and the group discussion tasks and visuals were also tested with the cochairs of the conference and a group of experts, and the feedback was used to set clear discussion tasks and discussion aids. This approach aimed to prepare each session discussions by building on the collective knowledge from previous sessions and to effectively facilitate technical discussions despite complex multidisciplinary topics.

The meeting schedule was designed as follows: Ahead of each of the 3-hour virtual meetings, the organizing team prepared introductory talks to set the task of the day, defined discussion questions, developed visual aids, and designed the discussion process. During the session, the outcomes of discussions were recorded by facilitators and note takers on Miro boards that were used during the session. After each session, debriefs with breakout group facilitators reflected on the group dynamics and technical discussion. The organizing team used all this information to adapt and refine the preparation of the next session, including the discussion questions and discussion inputs on Miro. Moreover, after each session and debrief, the organizing team updated the concept map on a summary Miro board to capture the progressive discussions and made it available for asynchronous review and comments by conference participants.

Synthesis of discussions using thematic analysis by the organizing team led to the identification of 5 key areas for immediate action for the development of metrics to assess the burden of infodemics and associated interventions. They were summarized alongside a participant-generated list of proposed actions and concrete next steps for each area for implementation.

#### Second Approach: Using the Purpose-Outcome-Process Model for the Conference

The purpose of the meeting was to determine how to measure the burden of infodemics associated with the information mix people access and the associated drivers for people’s behaviors over time and to discuss new ways to characterize information exposure and health outcomes that support this measurement. The expected outcomes of the meeting were to synthesize collective feedback and arrive at concrete next steps on (1) a concept map on the main pathways on the wider effects of infodemics (individual, society, health system, and policy); (2) a list of principles for ranking and prioritization of concepts and indicators to be used; (3) a prioritized list of actions, study designs, and metrics that need development; (4) the establishment of collaborations to advance the work. Because the expected outcomes were ambitious for the planned total 12 hours of deliberations, careful consideration was given to how the conference outcomes could best benefit from the expertise of participating senior academics and policy makers.

#### Third Approach: Designing for Emotional Pacing, Engagement, and Behaviors Supportive of Desired Conference Outcomes

Experience from previous WHO infodemic management meetings has shown that infodemiology discussions often require a design that helps overcome barriers in differences in the language, terminology, and focus of the actions or aims of research between researchers from different disciplines and practitioners from different health programs or evidence-informed policy functions in health authorities [17,18,25]. Several meeting design features aimed to address this:

- The concept map and lightning talks by experts at the beginning of the day were used to highlight perspectives from different scientific disciplines or public health practice on the discussion task of the day.
- Facilitators of small group discussions were coached and provided with facilitator guides with prompts to help them move the discussion toward the task and were given a space on the discussion boards, where they could record suggestions tangential to the task at hand.
- The schedule deliberately emphasized more discussion time in smaller groups in comparison to in-plenary to allow for maximum participation and exchange of experience.
- The synthesis of collective discussion was used to prepare for the next session. This was a resource-intensive activity that aimed to learn as much as possible from participants, while keeping them interested, engaged, and motivated to provide further input in the next session.
- The organizing team reflected back to the group not only a technical summary of the discussions but also the observations on the discussions—for example, the development of a common understanding of vocabulary and small group identities.
- Because the discussions were highly technical and required intense engagement, breaks were designed to be playful. Music videos on the topics of public health and science were played at the beginning of the meeting and during breaks to set the tone of interactions at the conference.

### Profile of Participants

The 86 invited participants included academics and public health practitioners from 48 organizations, including voices from 28 countries across 18 time zones, with a 56%:44% gender split in favor of women (n=48 females vs n=38 males). In addition, 48 additional invited academics and policy makers were not available to participate. The conference participants were academics selected by the organizers for the relevance of their publication record in the past 2 years for the purpose of this meeting or practitioners who were working in health metrics, measurement, and health program implementation. The participants also included 5 observers from civil society and global public health implementing partners. Conflicts of interest were reviewed in accordance with WHO procedures for the management of the declaration of interest for expert consultations [26]. An extended conference-organizing team comprising 32 members was drawn from across the WHO, the US Centers for Disease Control and Prevention (US CDC), and the George Institute for Global Health (TGI), India. More information about the structure and methodology of the conference is detailed in Multimedia Appendix 1.

### Framing Discussions With a Concept Map of the Wider Impacts of Infodemics

**Figure 1:**
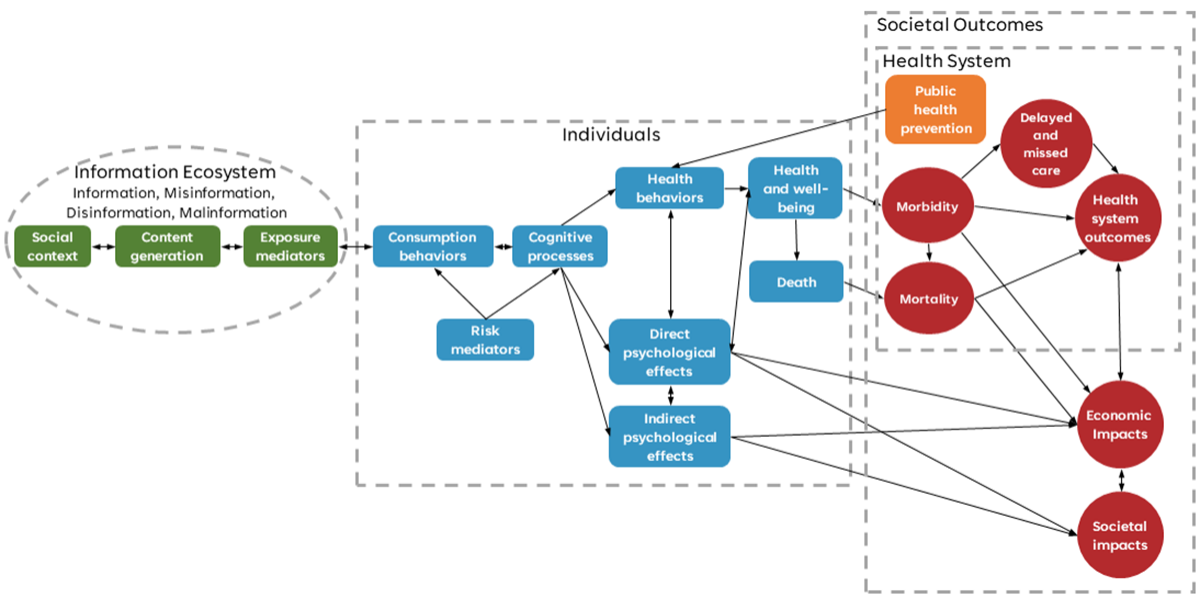
Concept map of the burden of infodemics, as discussed at the conference. It was organized across 4 domains: (1) in green, the level of the information ecosystem (online and offline content, social context, and structures that affect the dynamics of information consumption and transmission); (2) in blue, the individual level (behaviors and psychological mediators that determine exposure and susceptibility to the information characteristics of infodemics, as well as the proximal physical and psychological outcomes after this exposure); (3) in red, the level of health system impacts focused on metrics and outcomes specific to health care delivery and public health systems; and (4) in red, the societal level (infodemic impacts and ultimate outcomes that affect groups of individuals).

A map of concepts of the wider effects of infodemics was developed and used as a structured aid to facilitate streamlined discussions during the conference (Figure 1). The map itself was organized into 4 sections, representing elements relating to the hypothetical influence of the information environment and their potential effects on individual, health, and societal impacts. Further details of the burden of the infodemic concept map can be found in Multimedia Appendix 2.

Concept mapping is a technique from the social and natural sciences to represent hypotheses about how elements affect one another [27,28]. These maps are meant to be preliminary frameworks—for example, concept maps typically start in a highly qualitative form, similar to mind mapping or causal mapping techniques. Although concept maps may eventually inform the basis of quantitative research, such as structural equation modeling, highly qualitative concept maps can be helpful for nascent problems to provide a system-level visualization of potential causal links, which, in turn, informs strategies for their investigation.

A brief review of the literature did not yield any comprehensive existing frameworks to discuss the whole complexity of the infodemic. Therefore, a concept map was developed to help participants from different backgrounds have a common frame and vocabulary for discussion.

The draft concept map was based on theoretical expectations, drawing from existing models from multiple disciplines, including anthropology, psychology, sociology, and informatics. The concept map sought to apply exposure or dose-relationship models from medicine and public health toward infodemic impacts and drew from socioecological models to consider interactions between individuals and broader societal factors. It sought to provide a system-level visualization representing hypotheses about how key factors may affect outcomes in an infodemic. A synthetic map was needed as the majority of research to date has focused only on limited facets of the system. For example, 1 study sought to estimate the total monetized cost of decisions not to receive a COVID-19 vaccination based on misinformation or disinformation [16]. Another study focused on the incremental health costs due to additional COVID-19 cases caused by misinformation, as well as the impact on the gross domestic product due to government restrictions needed to address the infection growth rate attributable to the impact of misinformation [29]. Directionality and potential causal links between different concepts on the map would be a point of discussion during the meeting.

The concept map was designed to help overcome challenges associated with bringing together such a diverse group of participants from diverse fields and areas of public health practice and policy making. As research on infodemiology remains emergent, significant variations in how infodemics and their impacts are conceptualized exist. Any research seeking to measure the predictors, mediators, and impacts of either health behaviors or human cognition is intrinsically complex. The interdisciplinary nature of infodemiological research draws interest from a wide variety of diverse disciplines ranging from the social sciences to health informatics. Moreover, experts working in infodemiology vary in professional settings ranging from public health action to academic research.

The concept map was prepared by expert members of the organizing team, was shared with participants ahead of the conference, and was referred to through all deliberations. Several map limitations were communicated to the participants ahead of time. First, the map was used as a discussion tool, and its primary purpose was not considered a formal model. Second, elements that were likely to be challenging to measure were included in the map to foster discussion. Third, the model was based on theoretical expectations and not a systematic review of the literature. Fourth, the model was not comprehensive and should not be used to inform intervention design or quantitative modeling.

## Results

### Key Areas for Action

The meeting was oriented to formulate practical actions that could be taken in the future in the context that in November 2021, the COVID-19 pandemic continued to cause massive disruptions and the first countries were beginning to roll out COVID-19 vaccines as fast as possible. There were major concerns that the basic inputs that would underpin the burden of infodemic measurement were not yet in place, such as a common language, concepts, and thorough evidence and literature reviews. This was difficult to achieve due to the cross-disciplinary nature of the challenge. Therefore, practical, immediate actions were prioritized to strengthen the foundation for measuring the burden of infodemics.

There were many rich discussions on concepts and frameworks, and participants worked together to reach recommendations that would work toward coherence across disciplines. Together, we identified 5 key areas for immediate action toward the development of metrics to assess the burden of infodemics and associated interventions over the 4 sessions. The richness and evolution of discussions could not be fully reflected in the summary of the action areas, but we reflect on them broadly here. The concrete actions are summarized next, and more details of each of the action areas are provided in Multimedia Appendix 3.

First, participants noted that currently, although often referred to, no established and widely accepted definition exists of what exactly characterizes infodemics and related aspects (eg, misinformation) and thus urged to establish the development of standardized definitions related to infodemic measurement and management. This could be achieved through the establishment of a working group aimed at developing working definitions, which could later be validated using a Delphi method. Participants assessed this task as a priority since the term “infodemic” was conceptually conflated, was often overworked, and was currently used to refer to different concepts in different fields or country settings. A glossary of terms associated with the measurement of infodemics—examples include “information exposure,” “overload,” “risk mediators of individual effects,” and “delayed care due to infodemic”—with standardized definitions was urgently needed to aid infodemiology research as well as public discourse.

Second, participants proposed the establishment of a multidisciplinary working group to review and build on the concept map to reflect and reconcile different perspectives and disciplines that look at the information ecosystem, the individual, the health system, and societal factors contributing to the infodemic. A Delphi method was recommended to be used to validate the concept map. Efforts to improve the concept map should be closely coordinated with the technical working groups responsible for developing standardized outcomes (area 1) and with the group conducting a desk review of the evidence, tools, and data sources (area 3). This is essential as the definition of the appropriate elements in the map will be in association with the terminology being developed. Similarly, evidence from the literature reviews will be vital to arriving at relevant connections/associations between the elements in the map. Participants assessed this task to be a priority and voted to retain the infodemic burden concept map. However, participants warned against following any concept map too closely, as it might lead to disregarding critical elements that were not already elaborated on the map. They agreed on its value in identifying the various inputs and outcomes, as well as the confounding factors that determine the contours of a complex object of scientific inquiry, such as an infodemic.

Third, participants proposed the establishment of a working group to draft a protocol for conducting a review of evidence, tools, and data sources related to infodemic measurement. The working group would also explore options and partnerships that could implement the review. Participants assessed this task to be a priority. Given the emerging contours of infodemiology, its scope would extend beyond that of a traditional review. While drawing on tools for systematic reviews of ongoing and upcoming research, it would, for instance, also involve searches within the gray literature.

Fourth, participants suggested the establishment of a working group to review and improve different policy, practice, and research priorities on a rolling basis and work toward the alignment of infodemic management efforts at the global level by different stakeholders. Additionally, this group would support mainstreaming of infodemic management into public health practice, policy, and capacity building. This core group would be complemented by a wider array of related groups, leveraging expertise in specific areas in a Delphi method to reach consensus on various items discussed in the group. Participants assessed this task to be a priority.

Fifth, participants identified 4 urgent aspects of COVID-19 infodemic management needing attention in the short term. Additionally, participants ranked them in order of priority and offered inputs on their potential modification and expansion: (1) development of harmonized tools for the measurement of information diet/exposure and establishment of a global research collaboration to use them; (2) development of behavioral/process models that can be used for the development and evaluation of interventions; (3) measurement of the economic cost of the COVID-19 infodemic and related spill-over effects; and (4) identification of data sources and measures following the concept map, which can be used for defining global open data sets to facilitate modelling and research.

Participants agreed that the pandemic response, health system recovery, and resilience building remain key priorities for most health authorities and continue as a research focus for academicians. In addition to the 5 key areas of action, several additional themes of conversations were identified during the discussions (see details in Multimedia Appendix 4), in general reflecting on the barriers and enablers to assessing and measuring the burden of infodemics.

## Discussion

### Principal Findings

The meeting was started with the aim to discuss and arrive at a concrete action plan, but the discussion proved to be so rich that it was important to reflect on cross-disciplinary considerations for the burden of infodemic metric development. Therefore, the concrete action areas reflect the wider context that needs to be considered when discussing measuring the burden of infodemics. Participants reflected on the inherent tension between discussing abstract concepts and research gaps compared to the need to develop practical actions to move toward better measurement of the burden of infodemics quickly enough to assist in the current global crisis. Several considerations recurred in the discussions, cutting across all meeting days, which should be kept in mind when discussing frameworks for measuring the burden of infodemics.

- To successfully respond to infodemics and integrate infodemic management into health systems and health policies, it is crucial to be able to measure the burden of infodemics on society. The conference discussions reaffirmed that there is an urgent need for infodemiology research to be fast-tracked and oriented in directions that are most effective for infodemic management in public health. Efforts to identify metrics for assessing the burden and evaluating interventions related to infodemics will benefit if they proceed in a parallel manner. Metrics that are feasible to measure and implement across a wide range of public health programmatic settings should continue to be prioritized. Standard indicators already used for measuring health, population, and economy should be given priority over the invention of new ones.
- The identification of sources and metrics from established and routine health and data systems should be rigorously prioritized over the formulation of new ones. Integrating insights from online and offline sources of information would be essential to an objective infodemic burden assessment.
- Despite the efforts focused on characterizing misinformation, little research in the area has been designed to measure population-level associations between (mis-)information exposure and attitudes, such as vaccine hesitancy, or behaviors, such as nonadherence to public health practices [30]. Research in data-driven infodemiology has mainly focused on identifying the types of misinformation that appear online and their prevalence, often limiting itself to a single social media platform [31]. With a few exceptions [32], research designs do not associate information exposure with individual outcomes (eg, attitudes, practices, or behaviors) and thus cannot be used to assess the burden of infodemics [33,34]. This results in the absence of solid evidence that could support effective design of public health interventions.
- However, the difficulties in harmonized measurement of the burden of infodemics should not pause the efforts in public health practice to introduce evidence-based interventions through rigorous implementation research and adaptable health programming. For example, lessons should be drawn from how policies to address the burden of noncommunicable diseases on populations evolved over time. Measurements, such as monitoring of blood sugar levels, became standard practice and indicators before science was able to unequivocally link them to health outcomes and the burden of disease.
- Although the WHO Member States have recognized the perils of health misinformation [35], WHO, Member States, civil society, and other stakeholders have different roles to play in infodemic management and response. To be effective, management and response activities need to understand where the greatest risks are and rapidly capture the positive impact of responses without having to develop new, robust evaluation programs for every activity. Observational studies that simply report on the prevalence of misinformation make recommendations based on biased data and without measuring associations with behavior. For example, it was assumed that bots were important for disseminating misinformation, but research could not prove the real impact on the attitudes of social media users [36]. Studies that do not directly link information exposure to behavior can lead to wasted effort and unintended consequences. Understanding the mediating role that the social determinants of health play in individuals’ susceptibility to misinformation should be investigated.
- An infodemic causes harm on many levels, and it is by its nature a complex problem. Assessing its burden on health and society will require rethinking not only the frameworks, pathways, and protocols for measurement but also how the data are collected in a sustainable manner. WHO is developing activities to support pandemic preparedness and to mitigate the current pandemic, and several WHO preparedness activities rely on the development of new technologies and tools. The deployment of standardized tools for measuring how population-level differences in exposure to information risk factors explain the differences in behaviors after accounting for demographic differences is a challenge. New forms of global collaborations are needed to collect harmonized data through distributed collective measurement of the burden of infodemics. Moreover, research and data collection should consider using participatory research methods with communities and infodemic managers where the generation of metrics is paired up with interventions.
- Infodemics can be best addressed using a multidisciplinary approach and grounding in public health practice [17]. The currently emergent stage of the science of infodemiology, combined with the heterogeneity of academic expertise and professional backgrounds of the participants at the conference, offered rich opportunities for multifaceted technical discussions on metrics related to infodemics. At the same time, the meeting reconfirmed that conversations across diverse backgrounds must be prepared carefully to facilitate discussion across different scientific terminologies and approaches, as well as the differences between research methods and public health practice considerations.
- The lack of trust or mistrust toward health authorities can compromise adherence, compliance, and, ultimately, the overall success of the public health response, with all that these imply in terms of adverse outcomes on individual and population-wide levels. Identifying public health and social indicators for measuring and monitoring the impact of infodemics on health behaviors is now a priority for many health authorities that require evidence for planning, implementing, and evaluating interventions and policies. Trust metrics should be incorporated into infodemic metrics and modeling because these concepts are so interlinked.
- Currently, there are few published studies that reflect how policies foster or hinder infodemic-related outcomes; without measures that are identified that can be acted on by health systems, it will be difficult to institute more supportive and effective policies to mitigate the effects of infodemics on health.
- The way information access, exposure, and engagement are estimated for individuals is inconsistent across studies and often restricted to single social media platforms, limiting the value of the research. Furthermore, it remains unclear whether data from social media and web platforms can be used as proxy measures for a person’s broader information diet and whether these data capture differences in how people make sense of that information in terms of attention, trust, and prior beliefs. Ultimately, understanding how a person’s interaction with an increasingly individually attenuated and complex information ecosystem affects their health behaviors should be better studied to understand linkages to their online interactions.

However, there are ways forward to advance the measurement of infodemic harms and impacts and the use of infodemic management interventions. The 5 conclusions and 5 key actions from the conference represent the convergence of many of the limitations and opportunities mentioned before for the field and propose a roadmap for advancing the field for WHO.

### Other Policy Developments That Will Affect the Measurement of the Burden of Infodemics

Previously, many efforts in research and coordination in the misinformation space have focused on individual, societal, or media-related domains in a siloed manner. Now is the time to firmly center the health system in the infodemic management conversation when it comes to health emergencies. Strengthening preparedness, prevention, and resilience aspects to health systems in infodemic management will mean moving from defining terms and metrics to routinizing infodemic measures in routine data collection and decision-making in “peacetime” preparedness work and ramp up engagement, grounded in policy and enabled by sufficient workforce capacity and resources, during emergency activations of incident management structures.

In an attempt to reduce siloed approaches, multidisciplinary research and partnerships between public health, academic, media and civil society institutions should be fostered to identify interconnections which could provide basis for such assessments. Convenings similarly patterned on HCD principles may be well-suited to further discussing and establishing frameworks for interdisciplinary areas of health that are identified as priorities following emergencies and outbreaks, even if science and policy surrounding the topic is only emergent. This could include focus on burgeoning areas of governance, privacy and ethics in infodemic management and even in other health areas affected by infodemic harms. For example, WHO is convening a WHO ethics panel to deliberate on ethical considerations of social listening and infodemic management.

Ultimately, a successful infodemic response will lead to informed policies and promote healthy behaviors by individuals and communities. To do this, it identifies and addresses individuals’ and communities’ questions, concerns and information voids on health topics; reduces the spread and impact of misinformation; and refines public health engagement strategies (ie, promoting health equity, addressing scientific uncertainty and promoting culturally relevant risk communication and education) and health system response to more effectively promote healthy behaviors. To support countries, WHO has fostered development of tools to provide an evidence-based response to the infodemic and strengthen epidemic and pandemic preparedness activities [37,38]. These complement efforts by governments, media and factchecking organizations, civil society organizations and academic groups to develop valuable tools and resources to develop stronger methods for evidence-based decision-making for infodemic management. As the COVID-19 response has shown, all emergencies and pandemics in the future will be accompanied by infodemics that will be better addressed with the tools and insights developed today.

Health authorities seeking instructive policies or global technical guidance on infodemic management as the global epidemiological picture changes. WHO is working to establish a technical working group to support development of technical guidance that will be relevant to different country contexts, emergencies and outbreaks. A policy brief for COVID-19 infodemic management has also been published, outlining key recommendations for policy makers to integrate infodemic management in COVID-19 response and strengthen preparedness for other emergencies [39].

Countries are seeking solutions—interventions to stem current and future infodemics. Since the conference, WHO has commissioned an evidence gap map (EGM) exercise to analyze and visually map areas where there is evidence, the strength and applicability of that evidence of infodemic management interventions in the time of COVID-19 to the wider field, and where there are evidence gaps [40]. In conjunction with the conference outcomes and priorities identified by participating experts, this EGM can aid in prioritizing where investments in research and interventions should be directed.

### Conclusion

Infodemics now constitute a condition of our times and are here to stay, even it is extremely difficult to measure them precisely. To advocate evidence-based interventions for use in preparedness, prevention, and emergency response, a thorough assessment of infodemics’ impact and burden on society is required. This, however, requires to first reach consensus about what we exactly mean when we talk about infodemics and also about their moderating determinants. When definitions are set, formulating an adequate methodology—relevant in various health care settings and contexts—can be pursued that helps measure and eventually express the damaging effects of infodemics by using standard indicators. This conference was the first global step toward achieving these objectives.

We are standing on the shoulders of giants as diverse knowledge can be transferred from other disciplines and contexts into infodemic management for emergencies. Yet, we need further research and innovation to address some of the longstanding questions and bring about a truly multidisciplinary effort that serves both academic research and public health emergency preparedness, prevention, and response.

## Appendix

Multimedia Appendix 1 Conference meeting: methodology and structure.

Multimedia Appendix 2 Concept map.

Multimedia Appendix 3 Discussions about action areas.

Multimedia Appendix 4 Additional themes of conversations identified during the discussions.

## Abbreviations

HCD: human-centered design
US CDC: US Centers for Disease Control and Prevention
WHO: World Health Organization

## Data Availability

Raw notes from the conference proceedings are stored offline and are available upon request. Cleaned up versions of notes that were also shared with participants are available in the appendices.
