# Supplementary material for: Measuring the Burden of Infodemics: Summary of the Methods and Results of the Fifth WHO Infodemic Management Conference
Source: JMIR Infodemiology. 2023 Feb 20;3:e44207. doi: 10.2196/44207 (PMC9989916; doi:10.2196/44207)
Supplement: Multimedia Appendix 1 [file infodemiology_v3i1e44207_app1.docx]

## APPENDIX 1

## Conference meeting: methodology and structure

#### How the conference was run

The virtual conference took place during four three-hour working sessions on 2, 4, 9 and 11 November 2021. Each three-hour session was chaired and directed by two cochairs and followed a similar structure, first setting the scene of the day’s discussions in the plenary, then discussing in smaller groups, and then returning to plenary to reflect on collective outcomes. The core organizing team worked with 13 experts, who each prepared a lightning talk for one session to frame the discussion of the session day. The talks aimed to share experience and the insights from conducting research into various aspects of the infodemic, or from responding to the same from a public health standpoint. This was followed by breakout discussions, where participants brainstormed in smaller groups to respond to a set of technical tasks outlined on a shared Miro board (interactive virtual whiteboard) [25]. Breakout group discussions were supported by facilitators who were provided with facilitation refence materials and example prompts to elicit expert discussion before the discussion. Notetakers assisted in capturing major points of discussion and shared links and resources. Chat features on Zoom and a WhatsApp group were additionally available to participants to share insights, responses and views on the meeting proceedings during and between sessions, and larger emerging developments around infodemic management. The highlights and outcomes of each discussion were briefly reported back in the plenary by the group facilitators, so that all participants were abreast of significant observations.

#### Day 1

The concept map was introduced by members of the organizing team during the plenary session. It was subdivided into six focus areas to facilitate discussion among participants:

- Information landscape (social context, content generation, exposure mediators)
- Risk mediators
- Direct physical and psychological and indirect psychological effects, and health and well-being
- Health system outcomes
- Societal impact
- Economic impact.

Participants were also accordingly assigned to one of the six breakout groups, denoted by Groups A through F. Each breakout group discussed two parts of the concept map for 20 minutes each, where participants suggested concepts that fell into part of the assigned concept map.

After the initial 20 minutes of breakout discussions, groups were switched to different breakout rooms to discuss another focus area for 20 minutes. Given the rotation, the second focus area discussed by each group had already been the focus of another group’s discussion for the first round of 20 minutes.

Observations by group members were noted on the Miro board’s virtual sticky notes by an assigned note-taker in each group. Participants were free to directly add their own sticky notes to the board.

#### Day 2

The breakout group discussions during the second session of the Conference were also focused on the concept taxonomy map. Participants were assigned to the same group as on Day 1 to promote collegial discussion.

The following prompts were used to guide the group discussions in keeping with the session’s theme of “How do we measure information diet and information exposure?”

- What are the barriers and limitations that make it difficult to measure?
- What are the measurement tools that can be used or need to be developed?
- What study designs can be used to measure this?
- What indicators could be used or developed, keeping in mind that priority should be given to existing/routine data sources to ensure sustainability and feasibility even in low-resource settings?

#### Day 3

Breakout group discussions during the third session were organized around the theme of “Identifying measures for each of the concept taxonomy elements”.

Based on feedback from previous days’ sessions, the organizing team edited the notion of a concept map, which had initially been presented as a causal map, to a concept taxonomy. Through this modification, the various elements that needed capturing through existing and future metrics were prioritized instead of characterization of the nature of the logical relations between two or more elements.

There were four breakout rooms, each assigned to a specific theme of discussion towards the identification of available and potential measures around each of the four areas of the concept map. These were:

- information ecosystems;
- individual-level concepts;
- individual-level health outcomes;
- societal (including health system) outcomes.

Participants were free to join rooms of their choosing based upon their interests. They were also free to change rooms at any point during the discussions.

#### Day 4

During the final day of the Conference, participants were invited to join any breakout room of their choice. There were five breakout rooms, each dedicated to a specific task in view of the next steps to develop metrics and associated interventions for infodemic management.

- Develop standardized definitions.
- Improve the concept map.
- Conduct a desk review of the evidence, tools and data sources.
- Set up a technical working group.
- Address immediate priorities for COVID-19 recovery and resilience building.
