# Supplementary material for: Measuring the Burden of Infodemics: Summary of the Methods and Results of the Fifth WHO Infodemic Management Conference
Source: JMIR Infodemiology. 2023 Feb 20;3:e44207. doi: 10.2196/44207 (PMC9989916; doi:10.2196/44207)
Supplement: Multimedia Appendix 2 [file infodemiology_v3i1e44207_app2.docx]

## APPENDIX 2

## Concept map

The highly interdisciplinary nature and the currently nascent stage of the science of infodemiology, combined with the heterogeneity of academic expertise and professional backgrounds of the participants, offered rich opportunities for multifaceted technical discussions on metrics related to infodemics. These same features also presented significant challenges in terms of establishing a common language and framework needed for streamlined and actionable discussions around the measurement of the health effects of infodemics.

A concept map was introduced during the first working session of the Conference to facilitate discussions among participants on the technicalities of developing metrics for infodemics.

##### Why start with a concept map?

As research on infodemiology remains nascent, there are significant variations in how infodemics and their impacts are conceptualized. The reasons for this are fourfold:

1. The interdisciplinary nature of infodemiological research draws interest from a wide variety of diverse disciplines ranging from the social sciences to health informatics.
2. Experts working in infodemiology vary in practice settings, ranging from public health action to academic research.
3. Brief reviews of the literature suggest that comprehensive frameworks for the impacts of infodemics do not exist.
4. Any research seeking to measure the predictors, mediators and impacts of either health behaviours or human cognition is intrinsically complex.

As potential impacts will likely vary from the effects on individuals to broader societal-level outcomes, a lack of consensus will hinder discussions on how to think about infodemics and measure their impacts. A draft concept map would consequently facilitate conversation during this Conference.

##### What was the concept map used for?

Fig. 1 is a fictitious example based on world experiences from the COVID-19 infodemic, and its interactions between health authorities, media channels and sources, how infodemics affect individuals, families and communities, and possible outcomes.

In this concept map, boxes represented thematic constructs or elements hypothesized to be significant predictors of infodemic outcomes. Arrows suggested causality or influence in the direction indicated. Circles were thematic outcomes of interest to public health. Sticky notes designated ideas developed during the Conference and in small breakout groups.

While the same map was used over the three sessions, participants were given distinct prompts for discussion and actions related to the concept map, and towards the identification of metrics for infodemiology.


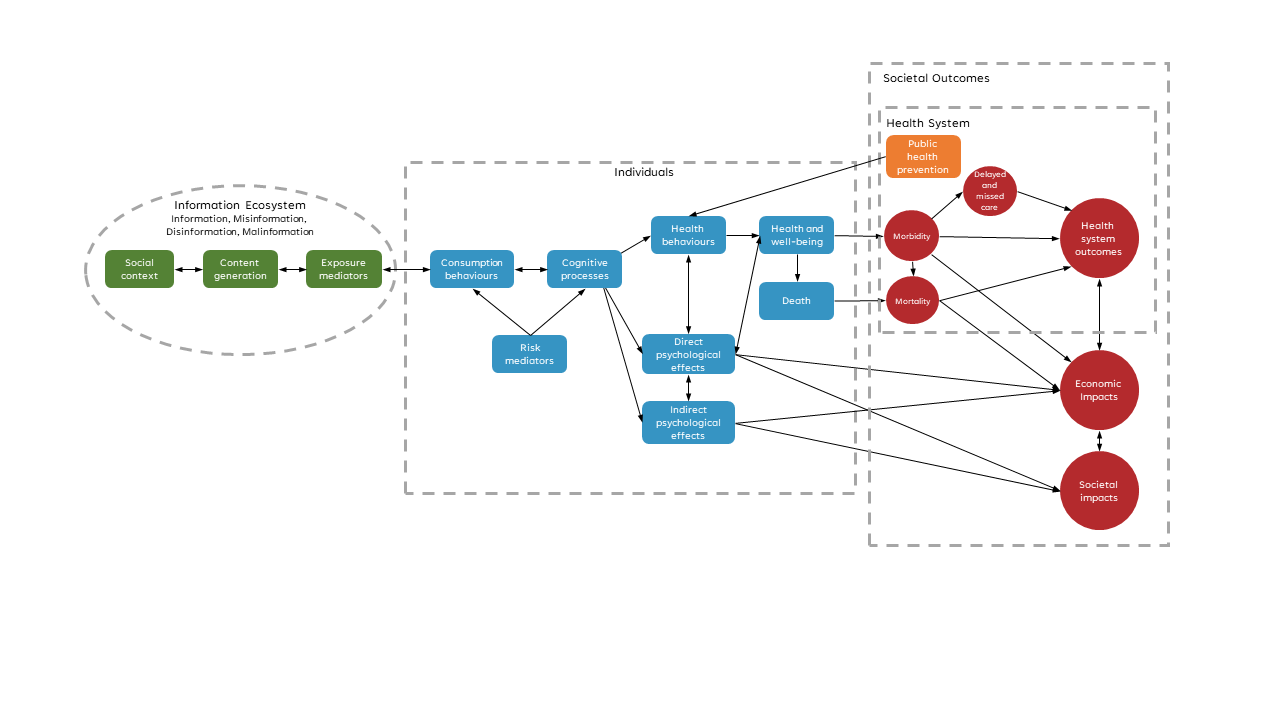


The map itself was organized into four sections, representing elements relating to the influence of information dynamics, and their attendant effects on individual health and societal impacts.

1. Information ecosystems: online and offline content, social context and the structures that affect dynamics of information consumption and transmission
2. Individual effects: behaviours and psychological mediators that determine exposure and susceptibility to information characteristics of infodemics, as well as the proximal physical and psychological outcomes after this exposure
3. Health system impacts focused on metrics and outcomes specific to health-care delivery and public health systems
4. Societal impacts: infodemic impacts and ultimate outcomes that affect groups of individuals.

In this concept map, boxes represented thematic constructs or elements hypothesized to be significant predictors of infodemic outcomes. Arrows suggested causality or influence in the direction indicated. Circles were thematic outcomes of interest to public health. Sticky notes designated ideas developed during the Conference and in small breakout groups.

##### How was the concept map used?

Different parts of the concept map were discussed over the course of the Conference in two formats: by discussants who were experts at providing perspectives on specific topics in plenary, and in small group discussions in breakout groups where all participants were asked to provide inputs on the topic.

The interactive Miro platform was used to present the concept map and capture inputs from discussions. Participants in breakout groups were encouraged to populate and edit the concept map, as per the specific facilitation instructions for the discussions. Alternatively, participants could either voice their position or make a note on the chat feature of the breakout room, and an assigned group discussion facilitator would capture the conversation through virtual sticky notes on the Miro board.

Some leading questions for the breakout groups in discussing the concept map are given below:

- What subconcepts should be added within the box on the map?
- What measures can be used to measure the concept?
- How can we prioritize adding concepts that are measurable and for which data sources may already exist?
- What are the barriers or considerations that make this harder or easier to measure in high-income countries? In LMICs? Globally?
