# Supplementary material for: Measuring the Burden of Infodemics: Summary of the Methods and Results of the Fifth WHO Infodemic Management Conference
Source: JMIR Infodemiology. 2023 Feb 20;3:e44207. doi: 10.2196/44207 (PMC9989916; doi:10.2196/44207)
Supplement: Multimedia Appendix 3 [file infodemiology_v3i1e44207_app3.docx]

## Appendix 3

## Discussions about action areas

Five key areas for immediate action towards the development of metrics to assess the burden of infodemics and associated interventions were identified over the four sessions. Participants discussed in detail the technicalities involved in implementing each, which are captured below.

#### 1. Develop standardized definitions

Participants agreed that this task was a priority because at present the term “infodemic” is used to refer to many scenarios and situations. As such, it is conceptually conflated, overworked. A glossary of terms associated with infodemics – such as misinformation, disinformation or an overabundance of information – with standardized definitions was sorely needed to aid infodemiology research as well as public discourse.

Several nuances around this task were anticipated, which would need to be addressed by the working group. These included the following:

- Apart from mis- and disinformation, how should notions that such information is/was true at one point in time but has become outdated/expired in face of newer evidence, be captured in the terminology?
- Should there be a term to indicate information that is not mis- and disinformation, i.e. so-called “good” or “true” or “accurate” information?
- Should infodemics, with regard to its connotations of “excess information”, be defined as a universally negative phenomenon? Where does this leave contexts in which “more information” might be adding value and fostering education rather than contributing to the reverse?
- Strong operational definitions of terms such as “overabundance of information” would be essential to develop operational metrics to measure infodemics, given the inherently relational and contextual nature of such concepts. What should be the markers of “overabundance” and what is the overabundance relative to? Is it fair to assume that individuals and societies are always overwhelmed with information?
- Is the idea of “overabundance” better applied to the magnitude of information to which people are exposed, or to the harms that are then caused? Harmful outcomes have the advantage of being easier to measure (in terms of mortality, vaccine refusals, and so on) but run the risk of abandoning the link with information exposure. Should the current definition be modified to “an overabundance of harm created through exposure to mis-, dis-, and an excess of information”?
- The question of language and the power of the translator to dictate the narrative. Along with a glossary, WHO should develop guidelines on how to translate the same into different languages. There may be many terms in a language that may have approximately the same meaning, but with different political connotations.
- Multiple terms that have approximately the same meaning are currently in use to describe infodemic-related phenomena. The duplication that happens when multiple terms are commonly used to denote essentially the same concept/ phenomenon adds to the confusion. A timely example would be “vaccine hesitancy” versus “vaccine confidence”. Another would be differences in meaning between “vulnerability” or “susceptibility” in risk assessments of mis- and disinformation.
- WHO should take the lead in producing a standardized glossary that can be used to inform scientific research, technical discussions and public discourse.

#### 2. Improve the concept map

Participants agreed that this task was a priority and voted to retain the infodemic burden concept map. They agreed on its value in identifying the various inputs and outcomes, as well as the confounding factors that determine the contours of a complex object of scientific inquiry, such as an infodemic. However, participants warned against following any concept map too closely, as it might lead to missing critical elements that were not already elaborated on the map.

A multidisciplinary steering committee should be formed to develop the concept map, and to identify stakeholders to define its elements and links. Efforts to improve the concept map should be closely coordinated with the technical working groups working on developing standardized outcomes (Conference outcome #1), and with the group conducting a desk review of the evidence, tools and data sources (Conference outcome #3). This is essential as definition of the appropriate elements in the map will be in conversation with the terminology being developed. Similarly, evidence from the literature reviews will be vital to arriving at appropriate causal connections between the elements in the map.

In terms of specific modifications, participants suggested the following:

- The map should be sensitive to the differences between mis- and disinformation and information overload, as these can lead to entirely different health behaviours and outcomes.
- Models on information-seeking behaviours should also be included in the map.
- Elements on the map should be refined so that they have the same level of granularity, instead of some elements being extremely broad and the others too narrow.
- Elements should also be reworked to make the model more broadly applicable to infodemics and health outcomes, rather than focus on a specific-use case alone, such as vaccination.
- A balance should be sought between making the concept map precise for a specific infodemic-related use case, which might help tailor better research questions, and its applicability for articulating general infodemic-related dynamics.

In terms of enhancing the utility of the infodemic burden concept map, participants made the following suggestions:

- There should be a clear understanding of and communication on who the target audience and what the target use for the map is.
- The map should be presented to select groups who did not participate in the Conference.
- A series of infodemic-related case studies, based on literature reviews, should be developed as use-cases to illustrate the analytical utility of the concept map.
- Training should be offered for different stakeholders (e.g. researchers and public health policy-makers) on how to use the map based on their specific research tools and needs. For example, the concept map might be integrated as part of the WHO [COVID-19 infodemic management course](https://openwho.org/courses/RCCE-COVID-19).
- Future meetings should devote more time to the revision and review of the concept map, with concrete-use cases to bring the discussions together.

In sum, the infodemic burden concept map could be envisioned as a living document that is refined and evolved as infodemiology research progresses and more is known about the links between information exposure and health outcomes. This would necessitate regular reviews with key stakeholders. The review committees should be diversely constituted to avoid fatigue and keep the advantage of fresh eyes to help identify different areas of focus where further research and measurement is needed.

#### 3. Conduct a desk review of evidence, tools and data sources

Participants agreed that this task was a priority. Given the emerging contours of infodemiology, its scope would extend beyond that of a traditional desk review. While drawing on tools for systematic reviews of ongoing and upcoming research, it would, for instance, also involve searches within the grey literature.

The process of compiling the desk review should be initiated by stakeholder mapping to identify networks in infodemiology research to link and reach out to. This could be followed by search query data for an idea of the data that is being collected but not shared widely, and a review of the public health interventions aimed at addressing infodemic burdens alongside a comparison with the infodemic burden concept map. Finally, experts and practitioners could be approached with additional interviews and/or surveys for a comprehensive idea of the evidence, tools and data sources currently in use.

A broad range of stakeholders should be invited to join and consulted in the desk review. Academics from across relevant disciplines, researchers at public health and communications and mass media institutes, representatives from consumer societies, social media platforms, community-based organizations, and social research institutions, including public opinion companies, should be invited. Consultants on the task might include area experts, policy-makers, health authorities, social media oversight boards, fact-checker organizations, marketing companies with a track record of studying messaging impact in terms of media and content.

In compiling a review of the existing evidence, tools and data sources being deployed in studies around infodemic burdens, particular attention should be paid to the following in knowledge synthesis:

- the choice and use of particular tools to investigate particular questions;
- the intersectional nature of the available evidence (from communication, marketing, public relations studies, and so on);
- the ways in which the digital divide (and the populations unreached by digital information) is being addressed;
- new and emerging data sources or methods;
- orioritizing evidence and measures that can be used to inform action, which are readily available, timely and associated with low costs of collection and a high confidence in the measure;
- the facilitation of a rapid critical appraisal of the evidence, tools and data sources;
- its translational aspects, focused on ways in which the nature of the information captured might be categorized and classified, so that it might be used by multiple audiences, including but not limited to policy-makers, health authorities, researchers and the lay public alike;
- its relevance at multiple levels – global, national and local;
- ways to maintain transparency and accountability with the technical working team.

A suite of complementary instruments would likely need to be developed to support the primary work of the desk review, including:

- standards to gauge and define the levels of confidence associated with specific evidence, tools and data sources;
- protocols for data-sharing, including standards for maintaining privacy/ anonymity, guidance on releasing metadata, or descriptive statistics, and coordinated negotiations with data providers;
- protocols and guidance on making databases interoperable;
- a validation exercise to facilitate the collation of a registry of validated data sources.

The desk review is an immensely collaborative task, which requires working with diverse data, languages and formats. Access to these data would likely be governed by a number of different legal frameworks that determine the ownership of data and its privacy protections. The willingness of various parties (governments, universities, technology corporations) to share relevant data might constitute another barrier. The participation of various stakeholders might be motivated by different incentive structures (non-profit organizations may want to raise funds around data findings, for instance), which must be negotiated.

A number of repositories and models – embodying specific principles, operating protocols and the objectives mentioned above – can be used as resources to inform this synthesis and review:

- Cochrane databases, for guidance on using standardized reporting and critical appraisals tools: <https://www.cochranelibrary.com>
- EQUATOR (Enhancing the QUAlity and Transparency Of health Research) network: <https://www.equator-network.org/>
- Global Research Map, which constitutes a worldwide landscape of digital health and artificial intelligence (AI) in health activity, methodologies and research via regional landscapes, done by iDAIR: <https://www.i-dair.org/launch-of-i-dairs-digital-health-and-ai-global-research-map-grm/>
- A repository focused on new and ongoing COVID-19 vaccine research in behavioural science, which was crowdsourced and curated by a non-profit research organization, without funding for the task: <https://docs.google.com/spreadsheets/d/1HrG2-ALI7Hku-T55jXGeCGm7DPsv30GOcymRnZYtZwg/edit#gid=45559234>
- CoVaxxy dashboard for misinformation on vaccines in the US, developed by the Indiana Universary Observatory: <https://osome.iu.edu/tools/covaxxy>
- Convening with newsrooms, journalists and health experts on the challenges of reporting on COVID-19 and vaccines in underrepresented communities: [Takeaways from the COVID-19 Community Convening](https://newsq.net/2021/09/16/takeaways-from-the-covid-19-community-convening/)
- Reaching offline communities, working with Google News Initiative to fund global fact-checking initiatives: [COVID-19 Vaccine Counter-Misinformation Open Fund](https://newsinitiative.withgoogle.com/covid-vaccine-counter-misinfo-fund/).

#### 4. Set up a technical working group

Participants agreed that this task was a priority.

A host of considerations should guide deliberations and decisions on the constitution, activities and expected impact of the working group:

- Include a wide network of individuals and institutions, representative of a wide range of actors and affected stakeholders, to minimize homogeneity and facilitate better advocacy and dissemination of work.
- Carefully select representatives from across the world and involve country-level practitioners, avoid overrepresentation by experts from areas with the maximum research funding and published literature on infodemic management.
- Include practitioners from the field to maintain focus on public health action and provide grounding on what is actionable, feasible and sustainable.
- Reach out to other working groups that are already working in this area and might be able to assist.
- Identify institutions at the regional and local levels (e.g. using the Vaccine Safety Network), whose work aligns with one or more of the proposed action areas to be part of the extended network.
- Ensure the technical and apolitical orientation of the group’s work to serve all organizations and the public.
- Commit to transparency in all decisions and processes of decision-making, facilitated by actions such as a public call for members, and a collaborative approach to setting up the terms of reference of the group.
- Look to international agencies to keep the focus on developing health-related metrics in infodemic burdens, while also deciding on appropriate limits to the notion of “health”.
- Specify concrete deliverables for the working group to facilitate timely assessments of its impact in advancing the field, relative to the time and resource investments incurred.

#### 5. Address immediate priorities for Covid-19 recovery and resilience building

Participants agreed that all aspects constituted essential work for developing metrics to measure the infodemic burden. A number of ways of ordering the priorities were put forth, subject to different lines of reasoning on why measuring the burden of infodemics constituted essential public health action.

The common priorities that emerged from the discussions, in rough order, are listed below:

1. Development of harmonized tools for measurement of the information diet/exposure and establishment of a global research collaboration to use them
2. Identification of data sources and measures following the concept map, which can be used for defining global open datasets to facilitate modelling estimation and research
3. Development of behavioural/process models that can be used for the development and evaluation of interventions
4. Measuring the economic cost of the COVID-19 infodemic and related spill-over effects.

Alternative priority actions on metrics development could involve the following:

- Developing measures to assess and capture public trust in institutions (in government agencies, health authorities, traditional media, social media, etc.), as well as in individuals (community-level leaders, influencers, etc.) and their relationship to infodemic and consumer behaviour
- Developing methods to track and measure how levels of trust change over time, subject to infodemic/risk communication interventions
- Identifying and developing measures to assess information voids, and to determine the costs associated with the voids as opposed to mis- and disinformation alone
- Developing metrics to describe and assess the consumption of infodemic diets and ecosystems, so that the notion of information exposure is well-defined
- Developing methods to capture cost savings made from investing in infodemic management as potential incentives for policy-makers, in addition to the economic cost burdens of infodemics.
