# Supplementary material for: Measuring the Burden of Infodemics: Summary of the Methods and Results of the Fifth WHO Infodemic Management Conference
Source: JMIR Infodemiology. 2023 Feb 20;3:e44207. doi: 10.2196/44207 (PMC9989916; doi:10.2196/44207)
Supplement: Multimedia Appendix 4 [file infodemiology_v3i1e44207_app4.docx]

## Multimedia Appendix 4

## Guiding principles for measuring the burden of infodemics

- The nature of the broader information ecosystem, particularly in its mix of sources and drivers of information creation, spread and consumption, necessitates the use of mixed-methods research in infodemiology, wherein qualitative aspects and methods of investigating infodemics are developed alongside quantitative metrics
- A human-centred approach should be adopted in developing metrics for infodemic research and interventions. This would call for participatory research methods and foreground the human subjects who are the intended beneficiaries of infodemiology research and health interventions.
- The effort of developing metrics to produce evidence of the health burden of infodemics should be calibrated from the health systems perspective of LMICs

## Developing ecosystems for data-sharing and knowledge synthesis

- It is essential to incorporate systems and platforms, and foster cultures of knowledge-sharing into infodemiology research, even as the scientific discipline is growing.
- While more research is undoubtedly required, there is an urgent need to focus on knowledge synthesis and promoting awareness of currently available tools as options for systematic implementation towards infodemic management
- To this end, there is a need to collate and build repositories of available information across datasets, sources, tools and policies being used for infodemic research and management (in line with Area #3).

## Putting infodemics on the health advocacy and public health action agendas

- Coordinated networks should be formed to engage a broad range of stakeholders to promote the visibility, awareness and action around the health costs of infodemics.
- These networks could also support countries and ministries of health in formulating policies and a governmental structure towards supporting infodemic management.

## Definitions and metaphors for metrics

Given that infodemics constitute complex objects of scientific inquiry, it is imperative to outline common conceptual frameworks and operational definitions to aid the development of metrics to ascertain their burdens. It becomes essential to reflect on the heuristic frameworks and terminologies in use to speak to and of infodemics, which will inevitably determine how they are measured.

- Standardized definitions to establish a common understanding of the various terms used to discuss infodemics would be a key step to measuring the health burden of infodemics (in line with Area #1).
- Understanding an infodemic as a one-off phenomenon, or one that is discrete and contains events that can be stopped, should be replaced with a recognition that infodemics are here to stay but can be managed and mitigated with the right strategies during an emergency or acute health event. An alternative analogy, for instance, might liken the health effects of infodemics to those of spurious treatment or mistreatment, instead of a disease.

## Measures for mitigation

Alongside developing metrics to capture the health burden of infodemics, we need to move towards building systems and societies that are resilient to infodemics, so that its burdens are minimized. Here, it is important to be mindful of the scope of infodemics; namely, that infodemics are not confined to mis/dis/information but are also about coping with an overabundance of information, which includes true and accurate information as well as questions, concerns and information voids. As infodemics now constitute a condition of our times and are here to stay, health education and early education on the various vectors of infodemics are crucial.

- There is an urgent need to integrate health literacy, including education, on how to search for and assess information from an early stage, just as the importance of healthy foods and healthy lives are now a routine part of primary education.
- There is a need to build an understanding among the public about the evolving nature of science and how public health recommendations are made and why they change.
- We need to be vigilant of the avenues and processes by which the output and evidence generated by research studies reaches the lay public and informs their health decisions.
